# Haplotype diversity of *VvTFL1A* gene and association with cluster traits in grapevine (V. *vinifera)*

**DOI:** 10.1186/s12870-014-0209-3

**Published:** 2014-08-05

**Authors:** Lucie Fernandez, Loïc Le Cunff, Javier Tello, Thierry Lacombe, Jean Michel Boursiquot, Alexandre Fournier-Level, Gema Bravo, Sandrine Lalet, Laurent Torregrosa, Patrice This, José Miguel Martinez-Zapater

**Affiliations:** 1 Instituto de Ciencias de la Vid y del Vino (ICVV), (CSIC, Universidad de La Rioja, Gobierno de La Rioja), CCT, C/Madre de Dios 51, Logroño 26006, Spain; 2 UMT Geno-Vigne® (IFV- INRA-SupAgro), 2 Place P. Viala 34060, Montpellier, Cedex 1, France; 3 Bio21 Institute, Department of Genetics, University of Melbourne, 40 Flemington road, Melbourne 3010, Australia; 4 INRA-SupAgro, UMR AGAP, équipe Diversité et Adaptation de la Vigne, 2 Place P. Viala, Montpellier 34060, Cedex 1, France; 5 CNB-CSIC, Dpto. de Genética Molecular de Plantas, Darwin 3, Madrid 28049, Spain; 6 INRA, Unité Expérimentale du Domaine de Vassal, Route de Sète, Marseillan-plage 34340, France; 7 current address: INRA, UMR Biologie du Fruit et Pathologie, B.P. 81, Villenave-d’Ornon 33883, Cedex, France

**Keywords:** Plant reproductive development, Inflorescence structure, Flowering time, Berry size, Grape domestication, Grapevine

## Abstract

**Background:**

Interaction between *TERMINAL FLOWER 1* (*TFL1*) and *LEAFY* (*LFY*) seem to determine the inflorescence architecture in *Arabidopsis*. In a parallel way, overexpression of *VvTFL1A*, a grapevine *TFL1* homolog, causes delayed flowering and production of a ramose cluster in the reiterated reproductive meristem (RRM) somatic variant of cultivar Carignan. To analyze the possible contribution of this gene to cluster phenotypic variation in a diversity panel of cultivated grapevine (*Vitis vinifera* L. *subsp. vinifera*) its nucleotide diversity was characterized and association analyses among detected sequence polymorphisms and phenology and cluster traits was carried out.

**Results:**

A total of 3.6 kb of the *VvTFL1A* gene, including its promoter, was sequenced in a core collection of 140 individuals designed to maximize phenotypic variation at agronomical relevant traits. Nucleotide variation for *VvTFL1A* within this collection was higher in the promoter and intron sequences than in the exon regions; where few polymorphisms were located in agreement with a high conservation of coding sequence. Characterization of the *VvTFL1A* haplotype network identified three major haplogroups, consistent with the geographic origins and the use of the cultivars that could correspond to three major ancestral alleles or evolutionary branches, based on the existence of mutations in linkage disequilibrium. Genetic association studies with cluster traits revealed the presence of major INDEL polymorphisms, explaining 16%, 13% and 25% of flowering time, cluster width and berry weight, respectively, and also structuring the three haplogroups.

**Conclusions:**

At least three major *VvTFL1A* haplogroups are present in cultivated grapevines, which are defined by the presence of three main polymorphism LD blocks and associated to characteristic phenotypic values for flowering time, cluster width and berry size. Phenotypic differences between haplogroups are consistent with differences observed between Eastern and Western grapevine cultivars and could result from the use of different genetic pools in the domestication process as well as different selection pressures on the development of table and wine cultivars, respectively. Altogether, these results are coherent with previous classifications of grapevine phenotypic diversity mainly based on cluster and berry morphotypes as well as with recent results on the structure of genetic diversity in cultivated grapevine.

## Background

Grapevine (*Vitis vinifera* subsp. *vinifera*) was domesticated in the Neolithic period (*ca*. 8500–4000 BC) [1] from wild populations of *Vitis vinifera* subsp. *sylvestris*[2]. Archaeological data traced back the location of the earliest evidence for large-scale winemaking, likely linked to the use of domesticated plants, to the north of Zagros Mountains and in the Caucasian region [3] around 6000–5000 BC which supports that geographic area as the location for primo domestication events. From there, grapevine cuttings were widely spread: first from North to South; and later from East to West around the Mediterranean basin pathway [3]. Vegetative propagation and dissemination, spontaneous events of hybridization among cultivars, breeding with local wild plants and likely secondary domestication events generated the pattern of admixture that is observed in current cultivars [4]–[9]. The use of different genetic pools along the process of grapevine domestication and human selection for different uses such as fresh consumption, raisin or wine production have resulted in large variation for cluster size, compactness and architecture among cultivars from different geographic locations [10].

The size and shape of grapevine clusters is determined by the development and growth of inflorescences as well as the efficiency of pollination, fruit set and berry growth. Generally, wine grape cultivars present small (150-250 g) and compact clusters with small berries, while table grapes generally have large (300-400 g) and less compact clusters with large berries. Some of them can even be extremely big weighting up to 1000-1500 g [11]. Negrul [12] distinguished different grape morphotypes based in part on cluster and berry traits. Cluster architecture has implications on disease susceptibility, since cultivars with compact clusters are more susceptible to rot by *Botrytis cinerea* than those of loose clusters [10],[13],[14]. In spite of the relevance of cluster structure and compactness, very little is known about its genetic control probably due in part to the complexity of the trait, which depends on many different variables along the growth of the plant as well as the environmental interactions during its reproductive development. There is a need to define cluster shape and size in terms of quantitative variables to understand its genetic determination. So far, only a few studies have tried to identify the main variables responsible for variation in bunch compactness in grapevine. In this sense, Vail and Marois [14] identified cluster weight as the main factor to explain its variation while Shavrukov et al. [15] proposed total cluster length and node number per rachis as two of the main ones. Recently, Tello and Ibañez [16] evaluated 19 indexes to estimate cluster compactness highlighting the role of various cluster parameters such as branch length and number. The study proposed a fast and good estimator for cluster compactness based on cluster weight and length.

Genetic and molecular analyses in model plants, such as *Arabidopsis thaliana*, demonstrated the interaction between *TERMINAL FLOWER 1* (*TFL1*) and *LEAFY* (*LFY*) [17],[18] in the establishment of inflorescence architecture. Their interactions supported a simple model explaining the evolution of plants inflorescence architecture [19]. *TFL1* belongs to a small gene family first identified in mammals as encoding phosphatidyl ethanolamine-binding proteins (PEBP) [20], which participates in a wide variety of biological functions in eukaryotes. In Arabidopsis, *TFL1* has been shown to function in the transcriptional repression of flower meristem identity genes [21]. *LFY* encodes a plant specific transcription factor [22], which serves as a flower meristem identity regulator activating the transcription of other flower meristem identity genes [23]. Recently, the existence of a common genetic pathway controlling inflorescence architecture in *Arabidopsis* and rice has been demonstrated indicating that this pathway could be highly conserved in angiosperms [24]. Following this report, four MADS-box genes are required to suppress *TFL1* in emerging floral meristems; what seems to be indispensable to initiate their differentiation.

In grapevine, the family of PEBP encoding genes includes at least five genes; three of them have deduced protein sequences related to Arabidopsis *TFL1*, being *VvTFL1A* the closest homologous sequence [25]. In fact, over-expression of *VvTFL1A* in transgenic Arabidopsis plants generates phenotypes of large and late flowering inflorescences reminding those observed when over-expressing the endogenous Arabidopsis gene [25]. Likewise, recent findings show that the extreme cluster proliferation and delayed anthesis observed in the reiterated reproductive meristems (RRM) somatic variant of grapevine cultivar Carignan was caused by a single dominant mutation in the *VvTFL1A* gene. This dominant mutation was identified as the insertion of a class II transposable element, *Hatvine1-rrm,* in the *VvTFL1A* promoter, triggering up-regulation of the corresponding *VvTFL1A* allele in reproductive and vegetative organs of the shoot apex [26]. These results suggested a role for *VvTFL1A* in the determination of inflorescence structure as well as on the branching pattern of the grapevine fruit clusters and the time of anthesis.

To further analyze the contribution of *VvTFL1A* to the phenotypic variation observed for reproductive and inflorescence traits in grapevine, the nucleotide diversity shown by this gene in a core collection of grapevine accessions was analysed and a candidate gene association approach on the variation observed for fertility index, phenological variables as well as several inflorescence and berry related traits was carried out. Herein the identification of *VvTFL1A* sequence polymorphisms associated with flowering and cluster traits is reported, being the most relevant ones corresponding to several INDELs in two intron regions. These INDELs are in LD with additional SNPs defining three LD blocks, which correspond to three major haplogroups. Interestingly, these haplogroups are characteristic of either wine or table cultivars in agreement with the cluster and flowering phenotype to which they are associated to.

## Methods

### Plant material

The plant material consisted of 140 grapevine cultivars corresponding to a core collection of *Vitis vinifera* L. subsp. *vinifera* intended to maximize agro-morphological diversity for 50 qualitative and quantitative traits [27]. All the cultivars are maintained at the INRA experimental station of Domaine de Vassal, Marseillan-plage, France (http://www1.montpellier.inra.fr/vassal/). The list of cultivars, pedigree when available, classification according to use (wine, table or wine/table), geographical group according to Bacilieri et al. [6], Lacombe et al. [28] and available data of the Vitis International Variety Catalogue (http://www.vivc.de/) are shown in Additional file 1. Classification according to Eastern and Western origin was obtained considering cultivars from the Iberian Peninsula (IBER), Western and Central Europe (WCEUR) and the Italian Peninsula (ITAP) as occidental cultivars; whereas cultivars from the Balkans (BALK), Russia and Ukraine (RUUK), Eastern Mediterranean and Caucasus (EMCA), Middle and Far East (MFEAS) were considered as oriental cultivars. For newly bred grape varieties, their pedigree was used to assess Western or Eastern origin to classify them according to their genetic origin and not according to breeding location. When genetic origin of pedigree was questionable, the cultivar was considered to present mixed origin.

### Phenotypic evaluation

Ten morphological traits related to the reproductive biology of grapevine were considered in this study. Among them, four were related to phenology (budburst time, flowering time, veraison time and maturity time); one to yield (fertility index); and five to berry and cluster features (berry weight and cluster length, width, weight and compactness). Cluster compactness was estimated from available data such as [cluster weight/(cluster length)^2^] [16]. Principal component analysis separated on axis 2 phenological traits from cluster size traits being fertility index opposite to all other traits on axis 1 (Additional file 2). Strongest correlations were found between maturity and *veraison* time (Pearson’s r = 0.82) as well as between cluster weight and width (Pearson’s r = 0.80). All traits were scored at the Domaine de Vassal and were expressed as the mean value for five plants per accession analysed a maximum of three years following the recommended OIV descriptors as shown in Table 1[11]. Details of phenotypic values obtained for each cultivar are given in Additional file 3. The phenotype distribution for these traits within the core collection is provided in Additional file 4.

**Table 1:** List of traits analysed

**Trait**	**OIV code**	**Description**	**Units**
Budburst time	301	Mean budburst time compared with Chasselas cultivar of reference	Days
Flowering time	302	Mean flowering time (50% of open flowers) compared with Chasselas cultivar of reference	Days
*Veraison* time	303	Mean *veraison* time (50% of turn berries) compared with Chasselas cultivar of reference	Weeks
Maturity time	304	Mean maturity time compared with Chasselas cultivar of reference	Weeks
Yield = Fertility index	153	(Number of inflorescence / number of shoot ) per plant	Count
Berry weight	503	Average berry weight at maturity (20°Brix)	Gram
Cluster length	202	Average maximum cluster length at maturity (20°Brix)	Centimeter
Cluster width	203	Average maximum cluster width at maturity (20°Brix)	Centimeter
Cluster weight	502	Average cluster weight at maturity (20°Brix)	Gram
Cluster compactness		Cluster weight/(cluster length)^2^	Gram/cm^2^

### Genotyping

For each genotype, 3.6 kb of the *VvTFL1A* gene (GSVIVT01036145001, chr6_20199669-20203319, Genoscope 12X) were amplified and sequenced using primers listed in Fernandez et al. [26]. DNA was extracted from young leaves of each genotype as described in Adam-Blondon et al. [29]. Amplifications were carried out using Taq DNA Polymerase (Qiagen) as recommended by manufacturer. PCR products were treated with Exosap-IT reagent as recommended by manufacturer and sequenced at the Genomic Service of the Parque Cientifico de Madrid in an ABI prism 3730 (Applied Biosystems) DNA sequencer. Base calling, quality trimming and alignment of ABI chromatograms was performed using SeqScape v2.5 (Applied Biosystems). Sequence polymorphisms were manually verified to establish genotypes. The nomenclature system used to name polymorphisms corresponded to letters followed by numbers: single letter correspond to the involved nucleotide substitution using the IUB’s conventional nomenclature and “Ins” is used to designed INDEL; positive or negative numbers corresponded to polymorphism position from the first base of the “ATG start codon”. Linkage disequilibrium (LD) calculations between polymorphisms were carried out using the LD option implemented in TASSEL v.2.1 [30].

Molecular diversity parameter estimates were calculated using DnaSP v4.50.2 [31]. Per site nucleotide diversity (π) [32], Watterson θ estimate [33] and Tajima’s D [34] were calculated for the whole haplotype set and separately for the three structured sub-populations (K1, K2, K3).

### Association tests

**Figure 1:**
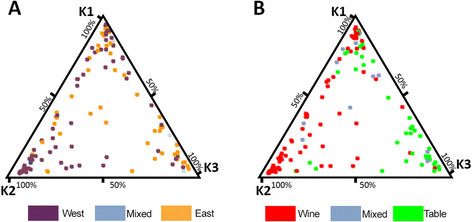
**Population structure of the** ***V.vinifera*** **core collection.** Schematic representation of estimated membership was obtained using STRUCTURE and classification of the individuals to one of the three genetic groups (K1, K2 and K3) was obtained using 50% of ancestry. Geographic origin **(A)** and fruit use **(B)** of each cultivar are indicated using colour codes.

Knowing that population structure can bias association studies, the structured association (SA) method [35] and the Mixed Linear Model MLM [36] were used to reduce false positives. Population structure of the core collection was determined using 20 SSR markers well scattered throughout the 19 grape linkage groups (LGs) [37] by a Bayesian clustering implemented in STRUCTURE v.2.3.4 [38]. The ADMIXTURE model was applied assuming that segregation of alleles was independent. A burn-in period of 100,000 followed by 150,000 Markov Chain Monte Carlo (MCMC) iterations with 5 replicate runs were carried out for each value of population structure tested (1 ≤ K ≤ 10). The optimal sub-population model was selected with the maximal likelihood *K = 3* according to the *ΔK* method [39] and later corrections for *ΔK* artefacts [40]. The corresponding Q-matrix was used for structured association tests. An arbitrary cut-off value of 50% ancestry was set to assign each individual to one of the three sub-population clusters (Additional file 1). Individuals not assigned in one sub-population were considered as admixed. This structure discriminates cultivars according to their use and geographic origin with K2 mainly constituted by western wine cultivars (78%), K3 by eastern table cultivars (78%) and K1 composed by eastern and western wine cultivars and table cultivars (Figure 1). The kinship matrix was calculated on the basis of the same set of SSR markers [41] using TASSEL v.2.1.

Comparison of the naïve General Linear Model (GLM) test, the structured association test (GLM-Q) and the structured Mixed Linear Model (MLM-Q) using TASSEL v.3 identified the last one as the most conservative model and was therefore selected to perform the association tests. MLM-Q association tests were carried out using the R v.2.15 [42] and TASSEL v.3 software. Polymorphic sites carrying rare alleles (frequencies <5% within the total sample) and unbalanced genotypic classes (frequencies <5% within the total sample) were discarded to avoid biased associations. Rare genotypic classes were in this last case replaced by missing data. Polymorphisms were codified to test both additive and dominant effects using R to be similar with marker model tested using TASSEL. For traits showing significant associations after Bonferroni correction (*P* ≤ 0.05) using either TASSEL or R, multi-locus mixed-models using forward-backward stepwise regression (MLMM) were implemented using the R software to identify major non-redundant associated markers [43]. Population structure and kinship were both included in the multi-locus analysis. Best models were selected according to the extended Bayesian information criteria (EBIC) and the multiple Bonferroni criteria (mBonf) according to Segura et al. [43].

### Haplotype reconstruction and networks

As *V. vinifera* genotypes are generally highly heterozygous [37], the unphased genotypic dataset was analysed to identify the succession of linked polymorphisms along the sequenced DNA region. Haplotypes were reconstructed using a PLEM algorithm [44] implemented in PHASE v2.1 applying default values of the iterative scheme [45]. Reconstructed haplotypes were submitted separately and simultaneously to three recombination detection tests implemented in the Recombination Detection Program v3beta41 [46]. Those were the MaxChi method with a window size of 12, 20, 25 or 30 variables sites [47], the Chimaera method with a window size of 12, 20, 25 or 30 variables sites [48] and the 3SEQ method [49]. To ensure consistency, haplotypes showing a significant probability of being the result of recombination (*P* ≤0.05) in at least two tests were considered as recombinants and excluded from further analysis as previously done by Fournier-Level et al. [50].

Network analysis was carried out using the median-joining method [51] implemented in Network v4.5.1.6 (Fluxus Technology, Sudbury, UK) and fixing a weight of 99 for the polymorphisms showing best associations with traits (Ins883, Ins422, K-737 and M-196). Three haplogroups HGA, HGB and HGC were defined according to the three LD blocks.

## Results

### *VvTFL1A* structure and sequence polymorphisms

**Figure 2:**
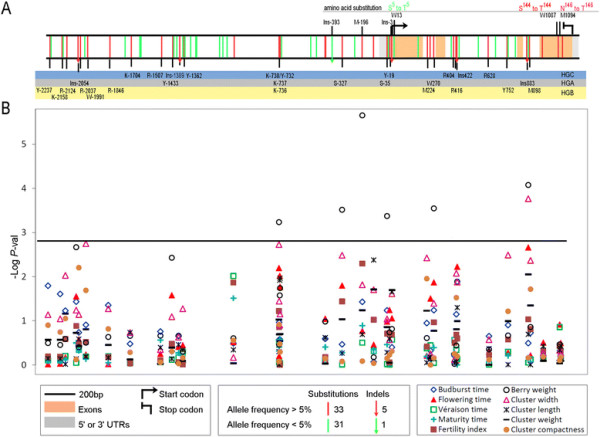
**Sequence polymorphisms identified for the** ***VvTFL1A*** **gene sequence and their association to phenotypic traits. A**. *VvTFL1A* polymorphisms found in 140 *V. vinifera* accessions. Single-nucleotide substitutions are depicted as vertical bars, different colours denoting allele frequency found in the collection (bottom legend box). INDEL are indicated as vertical arrows with similar colour codes. Amino acid changes are indicated. Polymorphisms are classified according to three LD blocks using a colour code: **B**. Level of structured MLM association detected between phenotypic traits and markers along *VvTFL1A* gene using TASSEL. *P*-value threshold 0.05 after Bonferroni correction is represented by the black line.

A total of 3646 bp of the *VvTFL1A* gene corresponding to 2442 bp and 1204 bp before and after ATG, respectively, were sequenced in all the individuals of the core collection. Translation of coding sequences identified the annotated four exons in the *V. vinifera* PN40024 genome sequence [52] of 201, 62, 41 and 218 bp and three introns of 83, 467 and 107 bp (Figure 2A). Nucleotide sequence analyses enabled the identification of 70 polymorphisms (64 SNP and 6 INDEL including 3 microsatellites). Among them, 44 polymorphisms were located in the promoter sequence, 4 in the 5’ untranslated region, and 5 and 17 polymorphisms were located in exonic and intronic regions, respectively. INDEL Ins-2054 and Ins-1389, located in the promoter, and Ins883 in intron 3, involved 5, 21 and 1 nucleotides, respectively; whereas Ins-393 and Ins-3 in the promoter, and Ins422 in intron 2 represented microsatellite variations with Ins422 being biallelic and the other two multiallelic (Figure 2A). Out of the five exonic SNP, only three (W13, W1087, and M1094) caused non-synonymous amino acid substitutions and are shown in Figure 2A. Among the 70 polymorphisms, 32 (46%) were represented by a rare allele (frequency <5%), with 10 of them grouped between position −1079 and −1430 before ATG. The complete genotypic data set is available in Additional file 5.

### *VvTFL1A* nucleotide diversity

**Figure 3:**
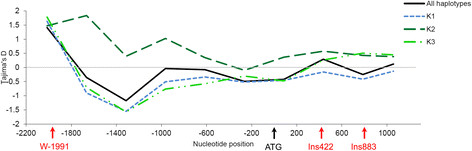
**Pattern of Tajima’s D values along** ***VvTFL1A*** **gene.** Neutral selection (D = 0) is represented by the grey line. Patterns have been obtained using sliding windows option from DnaSP (window length =500 and step size =350).

A total of 62 haplotypes, including 37 singletons, were identified based on phase reconstruction using the 70 segregating polymorphisms (Additional file 6). Genetic diversity of *VvTFL1A* gene was estimated in terms of number of segregating sites (S) and polymorphism (π and θ) for all the haplotypes and according to population structure. Comparison of genetic diversity index among the 3 genetic groups inferred within the analysed core collection (see Material and Methods) indicated that despite K3 sub-population included a smaller number of haplotypes than the two other K1 and K2 sub-populations; this presented a high number of segregating sites and π and θ polymorphism indexes (Table 2). The Tajima’s *D*-tests showed a general neutral value considering all haplotypes and a slight but non-significant negative value in K1 sub-population compared with K2 sub-population, which showed a slight positive value (Table 2). When Tajima’s *D*-test was estimated in sliding windows along *VvTFL1A*; the test revealed a similar pattern of variation for K1 and K3 sub-populations with a general negative value along *VvTFL1A* promoter (Figure 3). In contrast, the value of *D* along the transcribed region in K3 sub-population increased to reach a positive value. In K2 sub-population, Tajima’s *D*-test showed general positive value in both promoter and transcribed regions. These patterns indicated different selection or demographic events between haplotypes within the three genetic groups.

**Table 2:** **Pattern of diversity and neutrality tests for** ***VvTFL1A*** **gene**

	**All Haplotypes**	**K1**	**K2**	**K3**
S	70	62	50	48
H	62	37	35	16
π	0.00401	0.00365	0.00418	0.00412
θ	0.00439	0.00439	0.00348	0.00442
D_Tajima_	−0.29978	−0.61965	0.72764	−0.29739

S indicates number of segregating sites and H haplotype number. Analysis has been carried out in the three clusters of individuals related to the population structure. All tests yielded non-significant *P-*values (*P* >0.05).

### *VvTFL1A* haplotypes

**Figure 4:**
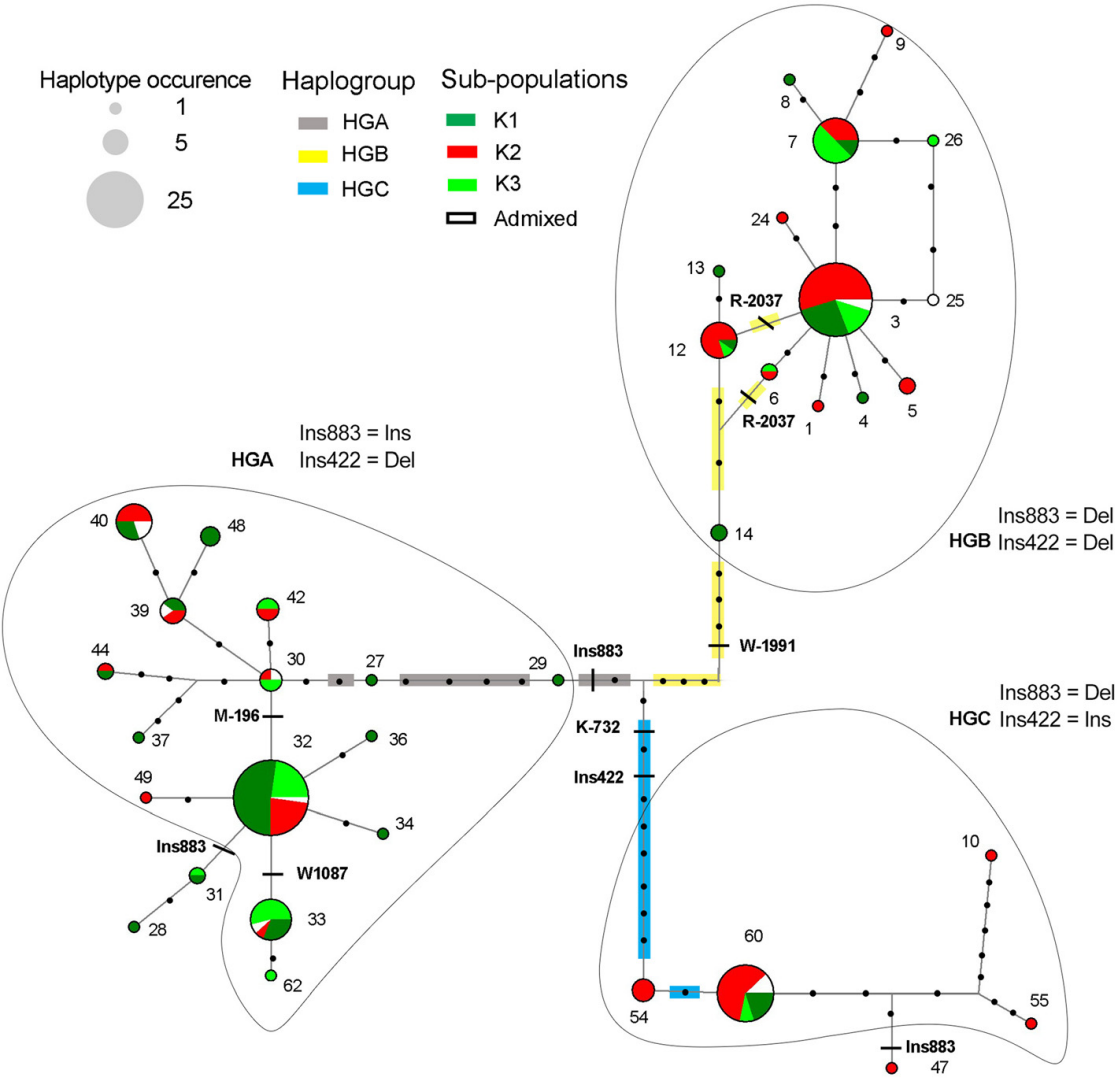
**Median joining networks derived from reconstructed DNA sequence haplotypes of** ***VvTFL1A*.** Network analysis was carried for haplotypes identified for 70 polymorphic sites in the *VvTFL1A* gene excluding recombined haplotypes. Haplotypes are represented by circles with circle size proportional to haplotype frequency (circle size corresponding to haplotype numbers of 1, 5 and 25 are illustrated). The haplotypes colours relate to the accessions classification according to according to structured genetic groups (Additional file 1). Black dots represent mutational steps. Colour lines represent the mutations in LD that separated the three haplogroups identified (HG).

In order to study relationship between *VvTFL1A* haplotypes, those likely resulting from recombinant events were detected to avoid bias. Among the 62 haplotypes originally identified, 26 were considered recombinants (Additional file 6) and were removed for haplotype network analysis. Haplotype network was constructed using 36 non-recombinant haplotypes that included 19 singletons. Haplotype network discriminated three groups of closely related haplotypes or haplogroups (HGA, HGB and HGC), which were clearly structured in relation with mutations in linkage disequilibrium (LD) (Figure 4). Within HGA major part of the haplotypes (44%) were present in cultivars of the K1 sub-population including Eastern and Western cultivars used as table and wine grapes. The remaining haplotypes of HGA were similarly found in cultivars of the K2 and K3 sub-population (25% and 24%, respectively). Regarding HGB and HGC 52% and 68% of their haplotypes were detected in cultivars of the K2 sub-population mainly represented by Western wine cultivars (Figure 4).

Haplotypes 32 and 3 were the most frequent (frequency >0.15 on the total haplotype pool and >0.19 when excluding the recombinants) and belonged to HGA and HGB, respectively. Most of the cultivars of the core collection were heterozygous for two different haplotypes (86%) with 20% of them being heterozygous for a combination of HGA and HGB haplotypes (Additional file 1). Only 20 cultivars were homozygous (14%) with eight and five varieties homozygous for HGA and HGB haplotypes, respectively. Two cultivars were homozygous for haplotype 54 of HGC and the remaining homozygous accessions presented putative recombinant haplotypes.

Regarding the recombinant haplotypes, haplotype 18, which was the most frequent (frequency = 0.05), corresponded to a recombination between haplotypes from HGA and HGB (Additional file 6). Indeed, no allele specifically assigned to HGC was present in this haplotype, which was always combined with alleles typical of both HGA and HGB haplotypes. Interestingly; haplotype 18 was present only in cultivars of K1 (40%) and K3 (60%) sub-populations classified mainly as Eastern table grapes, with two cultivars being homozygous for this haplotype (Additional file 1). Furthermore, among the individuals that presented at least one HGC haplotype mainly composed by cultivars of the K2 sub-population, the only one Eastern table cultivar belonging to the K3 sub-population was a combination with haplotype 18.

**Figure 5:**
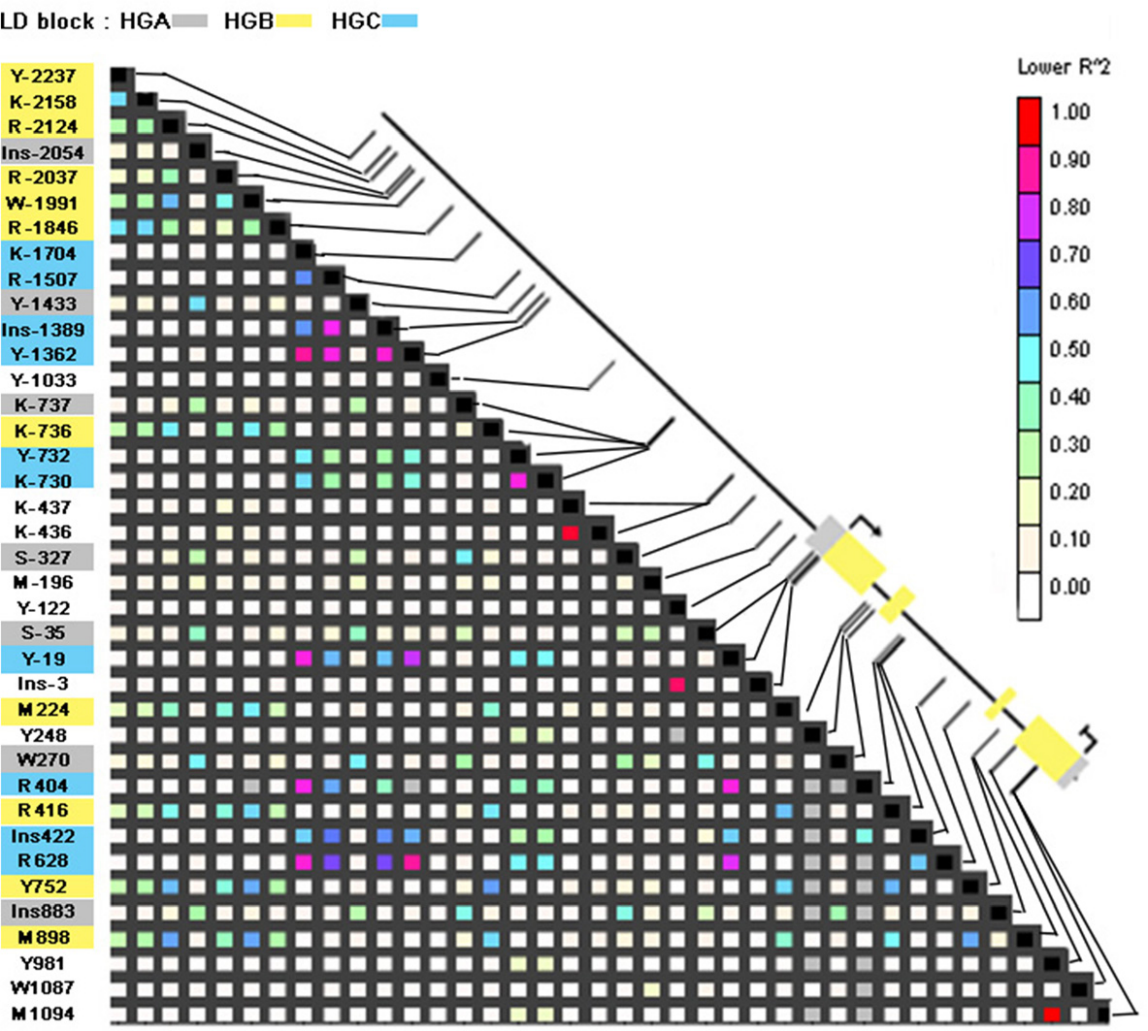
**Linkage disequilibrium among polymorphisms in the gene** ***VvTFL1A.*** LD plot based on R^2^ values for SNP and INDEL with frequency >5% were estimated according to Remington et al. [53]. The schematic representation of the *VvTFL1A* locus indicates ATG and stop codon position, exon regions represented by yellow boxes and UTR by grey boxes. Polymorphism classification into three LD blocks is represented by a colour code.

Certainly, LD pattern along *VvTFL1A* gene revealed three main blocks of linked polymorphisms (Figure 5): linked polymorphisms specific of HGA (Ins-2054, Y-1433, K-737, S-327, S-35, W270, Ins883) located in the promoter, the first and the third introns of *VvTFL1A* gene; linked polymorphisms specific of HGB (Y-2237, K-2158, R-2124, R-2037, W-1991, R-1846, K-736, M224, R416, Y752 and M898) identified in the distal promoter region and in the three intron regions; and linked polymorphisms specific of HGC (K-1704, R-1507, Ins-1389, Y-1362, Y-732, K-730, Y-19, R404, Ins422, R628) located in the promoter and the second intron of the *VvTFL1A* gene. Thus, haplogroups HGA, HGB, and HGC are consistent with the existence of three ancestral alleles or evolutionary branches supported by polymorphisms in the three LD blocks.

### Candidate gene association

Considering the biological function established for the *Arabidopsis TFL1* gene as well as the phenological traits altered in the Carignan RRM somatic variant, the candidate gene association study was focused on those phenological and cluster morphological traits that could be related with its putative biological function in grapevine. Association tests for *VvTFL1A* gene were run between the 38 polymorphisms showing minor allele frequency ≥5% and each phenotypic trait. Rare genotyping classes (≤5%) were excluded from the association tests. Out of the 38 polymorphisms, only 8 presented significant associations (adjusted *P-*value ≤0.05) using either R or TASSEL with flowering time, berry weight and cluster width (Table 3, Figure 2B).

**Table 3:** **List of** ***VvTFLlA*** **polymorphisms showing significant association after Bonferroni correction (<0.05) with flowering time, cluster width and berry weight through structured MLM tests using either R or TASSEL**

			**MLM-R**		**MLM-TASSEL**		
**Trait**	**Marker**	**Haplogroup**	***P-*** **value**	**Bonf corr < 0.05**	***P-*** **value**	**Bonf corr < 0.05**	**R2 Marker**
Flowering time	Ins883	HGA	2.72E-04	*	2.19E-03		0.104
	K-737	HGA	1.04E-03	*	6.42E-03		0.089
	Ins422	HGC	1.24E-03	*	6.01E-03		0.089
Berry weight	M-196		1.47E-08	*	2.23E-06	*	0.163
	Ins883	HGA	5.76E-07	*	8.40E-05	*	0.118
	S-327	HGA	1.33E-06	*	3.06E-04	*	0.105
	K-737	HGA	3.54E-06	*	5.83E-04		0.095
	S-35	HGA	9.17E-06	*	4.24E-04	*	0.105
	W270	HGA	1.02E-04	*	2.84E-04	*	0.112
	Ins-2054	HGA	1.58E-04	*	2.16E-03		0.074
	Ins422	HGC	2.32E-04	*	1.30E-02		0.059
Cluster width	Ins883	HGA	1.63E-05	*	1.72E-04	*	0.130
	K-737	HGA	9.18E-05	*	1.88E-03		0.097
	S-327	HGB	3.34E-04	*	3.30E-03		0.086
	Ins422	HGC	1.07E-03	*	1.34E-02		0.066

Corresponding *P-*value and variance explained by the marker (R^2^ Marker) obtained using TASSEL is indicated, *correspond to significant adjusted *P-*values(<0.05).

The strongest association was found between berry weight and SNP M-196 (*P* = 1.4E^−8^) explaining 16% of the trait variation. The highest association for flowering time and cluster width was found with Ins883 (*P* = 2.7E^−4^, *P* = 1.6E^−5^, respectively) that explained 10% and 13% of trait variation, respectively. Interestingly, Ins883 characteristic of HGA also associated significantly (*P* ≤0.01) with berry weight (*P* = 5.7E^−7^). At a lesser extent, Ins422 from HGC associated with the three traits explaining 9%, 6% and 7% of flowering time, berry weight and cluster width variations, respectively.

In order to determine whether the different associations detected were only due to LD or were the result of the particular effect of each polymorphism, the multi-locus mixed-model analysis was carried out. Flowering time showed the strongest associations with polymorphisms characteristic of HGA and HGC under single-locus approaches (Table 3). In the multi-locus analysis, the best models to explain flowering time variation identified one and two polymorphisms based on optimal mBonf and EBIC criteria, respectively (Table 4). The optimal models included Ins833 from HGA specific LD block and W1087 without LD with other polymorphisms and explained up to 16% of flowering time variation. Association between W1087 and flowering time was not identified with the single-locus approach; the use of Ins883 as covariate in the model revealed W1087 association. The remaining markers, not included in the model, had minor and/or redundant effects with those ones. Similarly, the best models explaining up to 25% of berry weight variation included M-196 and Ins883 in agreement with the highest associations detected with the single-locus approach. Instead, cluster width variation showing associations with polymorphisms within the three LD blocks under single-locus approaches was only explained by polymorphism Ins883 (HGA) after the multi-locus mixed-model analysis. The effect of polymorphisms characteristic of HGB was minor and redundant with those of Ins883. Therefore, in addition to its high association (*P* ≤0.01) in single-locus analyses with three traits (flowering time, cluster width and berry weight), Ins883 was selected in the three best multi-locus models explaining the variation of those traits. Besides, Ins883 explains alone cluster width variation. These results highlight the major influence of INDEL Ins883 in the possible role of *VvTFL1A* on phenology and cluster traits.

**Table 4:** MLMM results

**Trait**	**Forward step**	**mBonf <0.05**	**EBIC**	**Markers in the model**	**Gene region**	**Haplogroup**	**R2 Markers**	**R2 Model**
Flowering time	1	8.98E-03	609	Ins883	Intron3	HGA	0.11	0.19
	2	2.61E-01	603	+ W1087	Exon4		0.16	0.25
Cluster width	1	5.38E-04	492	Ins883	Intron3	HGA	0.13	0.34
Berry weight	1	1.26E-06	431	M-196	Promoter		0.21	0.76
	2	1.49E-01	412	+ Ins883	Intron3	HGA	0.25	0.80

The models presented corresponded to the optimal models, i.e., optimizing EBIC and mBonf criteria. R2 Markers = variance explained by polymorphisms in each model, R2 Model = cumulative variance explained by markers and genetic variance (structure and kinship).

### Phenotypic values related to major haplotypes

**Figure 6:**
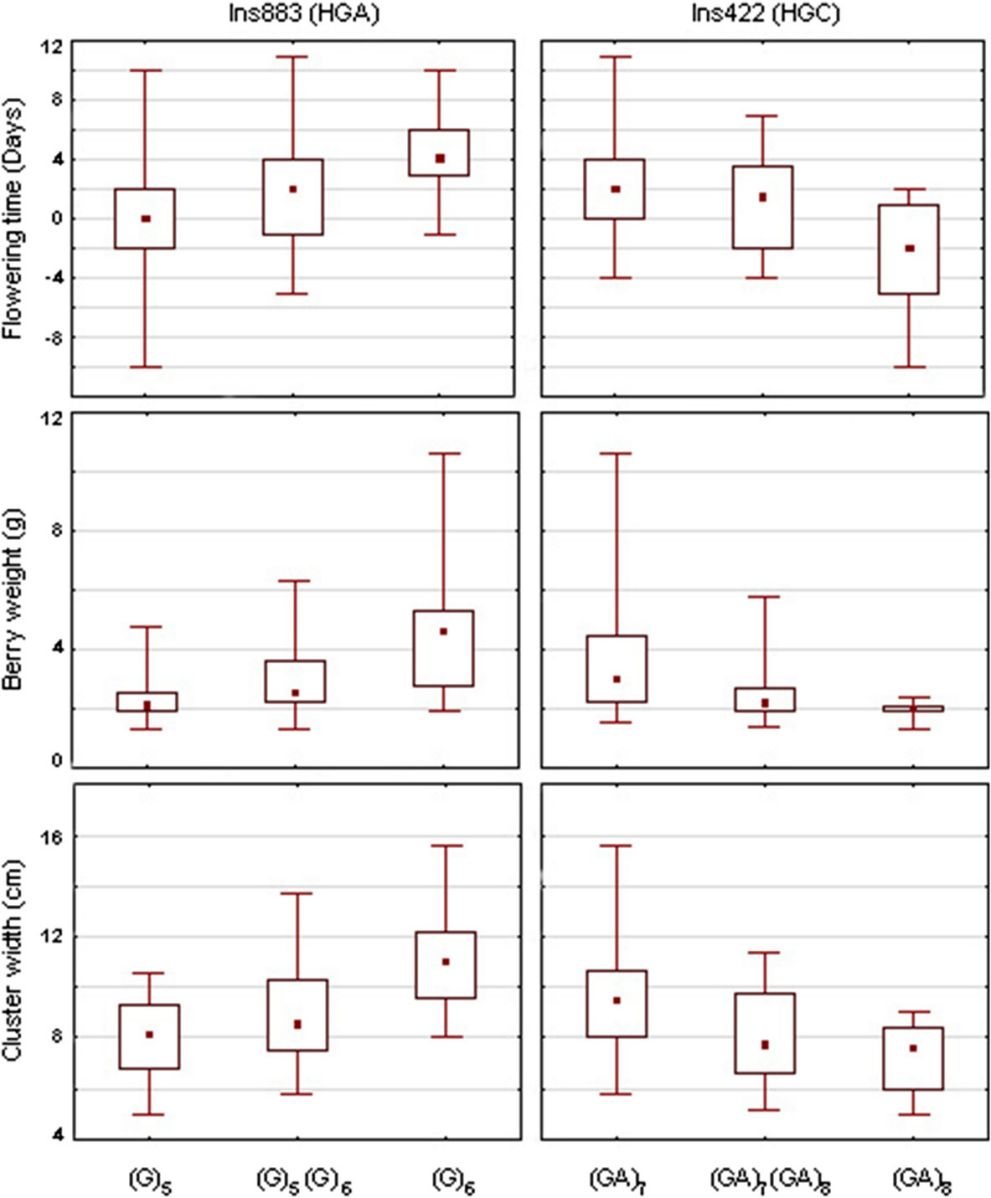
**Effects of** ***VvTFL1A*** **INDEL Ins883 and Ins422 on flowering time, berry weight and cluster width.** Box plots represent minimum and maximum (whisker), median (square dot), and 25th and 75th percentiles (box) values.

Based on haplotype network and association results, two molecular polymorphisms (Ins883 and Ins422) were selected that discriminated the three haplogroups (HGA: Ins883(G)_6_-Ins422(GA)_7_; HGB: Ins883(G)_5_-Ins422(GA)_7_; HGC: Ins883(G)_5_-Ins422(GA)_8_). The (G)_6_ allele of Ins883 was associated with late flowering time, high berry weight and large cluster width (Figure 6). All haplotypes in HGA presented the (G)_6_ allele, in contrast with haplotypes in HGB and HGC that contained the (G)_5_ allele Regarding Ins422, the (GA)_8_ allele was associated with early flowering time, low berry weight and small cluster width (Figure 6). All haplotypes carrying the (GA)_8_ allele belonged to HGC, mainly represented by haplotype 60, while haplotypes in HGA and HGB contained the (GA)_7_ allele.

**Figure 7:**
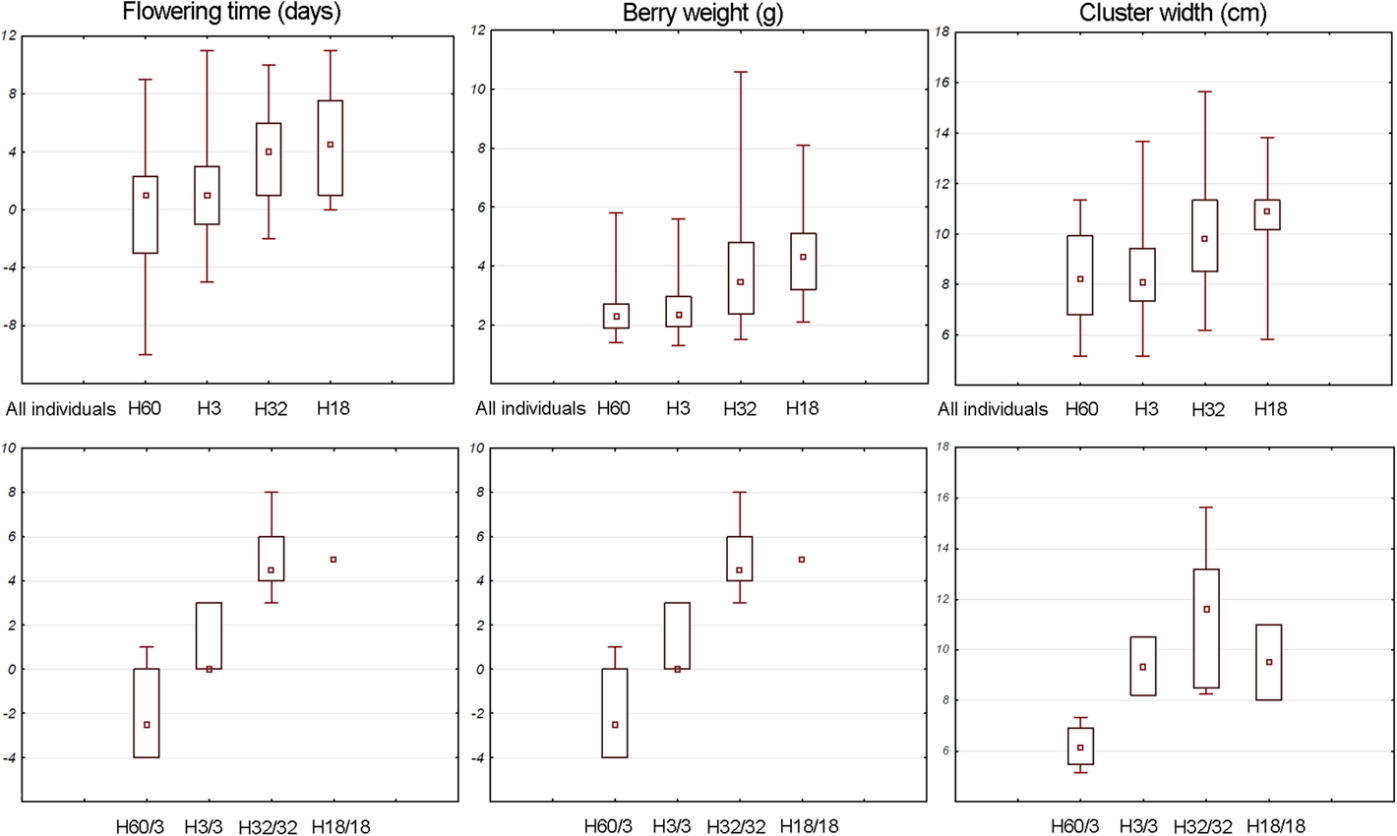
**Phenotypic value for flowering time, berry weight and cluster width related to major haplotypes.** Phenotypic values of individuals containing the major haplotypes H32, H3 and H60 and for the recombinant haplotype H18 at heterozygous or homozygous state. Box plots represent minimum and maximum (whisker), median (square dot), and 25th and 75th percentiles (box) values.

As other polymorphisms appeared associated with these phenotypic traits, the phenotypic values were also analysed considering the three major haplotypes 32, 3 and 60 from HGA, B and C, respectively. As an average, individuals presenting at least one haplotype 32 exhibited late flowering, big berries and large cluster clearly in contrast to the phenotypic features of individuals containing at least one haplotype 3 or 60 (Figure 7). This was more obvious when homozygous individuals or heterozygous individuals for haplotypes 3 and 60 were taken into account considering the absence of homozygous individuals for haplotype 60 (Figure 7). The phenotypic value for the more frequent recombinant haplotype 18 found in Eastern table cultivars was also analysed, which presented the (G)_6_ allele at the major INDEL Ins883 such as haplotypes of HGA. Phenotypic values for flowering time, berry weight and cluster width for haplotype 18 were slightly higher than those of individuals carrying haplotype 32 (Figure 7).

## Discussion

*Arabidopsis TFL1* plays a critical role in the specification of the inflorescence meristem and inflorescence architecture [54],[55]. This role seems to be conserved in other plant species [56] likely through a conserved regulatory pathway [24]. In grapevine, the previous identification of misexpression of the *Arabidopsis* homolog *VvTFL1A* as the molecular cause of the reiteration of reproductive meristems (RRM) mutant [26], also supported the possible conservation of its biological function in this species in agreement with previous results [57],[58]. Phenotypic characterization of the RRM plants showed that *VvTFL1A* overexpression was related to a delay in the time of anthesis and to an increase in the size and branching pattern of the inflorescences [26], similar to the effects of *TFL1* overexpression in transgenic Arabidopsis [21]. To provide additional evidence on the involvement of *VvTFL1A* in natural variation for flowering time and inflorescence development and to identify nucleotide sequence polymorphisms that could be partially responsible for those traits in grapevine, a genetic diversity analysis of this gene sequence and genetic association studies with those traits were carried out.

Nucleotide variations for *VvTFL1A* in the grapevine core collection analysed is relatively high with an average of one polymorphic site every 50 nucleotides. However, only five out of the 70 polymorphisms detected are located in exonic regions and only three of them result in non-synonymous amino acid substitutions. This result is in agreement with the slight negative Tajima’s D values observed along *VvTFL1A* coding sequences and suggests that the protein structure admits few variations. Reduction in overall level of nucleotide variation was also reported for the Arabidopsis *TFL1* gene when compared with other flowering genes [59]. Among the three non-synonymous polymorphisms identified in *VvTFL1A*, W13, located in first exon, had a very low frequency and was not considered for the association analyses. The two other, W1087 and M1094, are located in the fourth exon in a region of the protein responsible for the functional divergence between FT and TFL1 [60]; although the substituted amino acids do not correspond to conserved residues [60] and the SNP did not associate with inflorescence related traits under single-locus models. However, W1087 was selected by the multi-locus mixed-model analysis to explain part of flowering time variation together with Ins883 suggesting a possible functional effect of this SNP in this trait. Regarding the 17 polymorphisms found in intron regions, two INDEL (Ins422 and Ins883) showed significant association with flowering and cluster trait variation. INDEL Ins422 is located in intron 2 and corresponds to a microsatellite sequence of GA repetitions; while INDEL Ins883 is located in intron 3 and corresponds to a G nucleotide repetition. The 48 remaining *VvTFL1A* polymorphisms identified in the core collection were located upstream of the translation start codon and included four INDEL. No traces of the *Hatvine1-rrm* transposon were detected in the promoter of *VvTFL1A* in the whole core collection which demonstrates the specificity of the insertion event causing the Carignan RRM mutant phenotype [26].

Nucleotide polymorphisms in *VvTFL1A* LD blocks that discriminate the three haplogroups displayed differential association with cluster traits under linear regression models. Among all traits analysed, polymorphic sites characteristic of HGA and HGC haplogroups explained part of the phenotypic variation for flowering time, berry weight and cluster width. In the same way, polymorphisms specific of HGB associated with cluster width as well as polymorphisms from HGA. These results suggest that variation at *VvTFL1A* has an effect on flowering time, berry weight and cluster width with different alleles having differential effects on the traits. Interestingly, both flowering time delay and cluster width increase were observed in the phenotypic characterization of the RRM somatic variant related to *VvTFL1A* overexpression. Unfortunately, berry size was not measured in that study [26].

Among all the polymorphic sites tested, insertion Ins883 discriminating HGA from HGB and HGC explained alone part of flowering time, berry weight and cluster width variations. According to the multi-locus analysis, Ins833 explained up to 16%, 13% and 25% of flowering time, cluster width and berry weight variation in the best models, being the only polymorphism contributing to berry weight variation. INDEL occurring in functionally important regions of genes could affect gene function, through gene expression modification [61] or RNA structure alterations [62]. However, a preliminary *VvTLF1A* RT qPCR expression analysis carried out in young inflorescences of the cultivars of the core collection did not reveal any association between gene expression variation and the *VvTFL1A* polymorphisms (data not shown). Likewise, no clear correlation (Pearson’s r <0.28) between *VvTFL1A* expression and phenotypic traits was identified (data not shown). Nevertheless, these negative results do not discard a possible role of this intron sequences in transcriptional or posttranscriptional processes given the difficulties in carrying out transcriptional comparisons among different genotypes with different flowering behaviour. Analysis of maize *TFL1* homologs expression in different tissues and developmental stages showed the existence of differential transcript processing [63]. In fact, in a preliminary study, the existence of alternatively spliced RNA forms was detected for the first and the second introns of *VvTFL1A* (data not shown). Further research will be required to demonstrate any functional role of this alternative splicing as well as its relationship with the described *VvTFL1A* Ins833 polymorphism. In any case, further association analyses using larger samples and specific segregation analyses will be required to confirm the detected associations.

Together with Ins883, M-196 and W1087 without LD with other *VvTFL1A* polymorphisms explain part of berry weight and flowering time variation according to multi-locus analysis, respectively. In contrast to Ins883 that discriminates haplotypes of HGA from those of HGB and HGC, M-196 and W1087 corresponded to mutations differentiating haplotypes within the HGA haplogroup (Figure 4). The M-196 base change located in the proximal promoter and the W1087 non-synonymous substitution in the fourth exon of *VvTFL1A* might represent relevant structural modifications at the promoter and the protein sequence, respectively, likely affecting *VvTFL1A* function in a non-redundant way with Ins883. Moreover, in silico analysis using SIFT program (http://sift.jcvi.org/) predicts that substitution of T by S at position 144 of the VvTFL1A sequence affects protein function with a score of 0.04 based on the alignment of 240 closely related sequences.

*VvTFL1A* haplotype network differentiates three haplogroups of closely related haplotypes. Each HG is represented by a high frequency haplotype, haplotypes 32 for HGA, 3 for HGB and haplotype 60 from HGC. Consistent with the results of the association analyses, individuals containing haplotype 32 of HGA, exhibited late flowering, large cluster width and larger berries. Interestingly, most of the cultivars of K1 and K3 classified to table or table/wine uses, characterized by these phenotypic features [6], present HGA haplotypes. This relationship is also true for accessions carrying the recombinant haplotype 18. Indeed, haplotype 18 contains Ins883 insertion present in HGA haplotypes and mostly present in Eastern table cultivars belonging to K1 and K3 genetic groups. The fact that cultivars carrying haplotype 18 display late flowering, large cluster width and larger berries supports a clear relationship between Ins883 polymorphism and the eastern table cluster characteristics. In contrast, individuals containing haplotype 60 of HGC with Ins422 insertion exhibited early flowering, shorter cluster width and smaller berries. Consistently, HGC haplotypes are enriched in Western wine grape cultivars mostly belonging to K2 genetic group, which are known to display those cluster and berry features [6]. Finally, HGB haplotypes do not contain Ins422 or Ins883 insertions. The phenotype of individuals containing haplotype 3 (most frequent within HGB) is similar to some extent to that of cultivars carrying haplotype 60 (HGC). Consistently with this phenotype, HGB haplotypes are mostly present in cultivars for wine use. Because no homozygous individuals were observed for haplotype 60 in the core collection, the phenotypic effect of this haplotype is supposed to be much stronger in homozygous state, which suggests that haplotype 60, related to extreme phenology and cluster characteristics, could be less favored in cultivars in homozygous state than haplotype 3. Interestingly, partial sequencing of *VvTFL1A* in 20 *V.v* ssp. *sylvestris* plants from the Iberian Peninsula identified wild haplotypes similar to haplotype 60 and belonging to HGC (data not shown). These data could indicate a western origin for haplotypes of HGC.

## Conclusions

Three major *VvTFL1A* haplogroups were identified in cultivated grapevines based on the presence of three main polymorphism LD blocks. These haplogroups are associated to characteristic phenotypic values for flowering time, cluster width and berry size. Phenotypic differences between *VvTFL1A* haplogroups are consistent with the classification of grapevine phenotypic diversity in three different morphotypes proposed by Negrul [12] and could result from the use of different genetic pools in grapevine domestication and/or the existence of different selection pressures on the development of table and wine cultivars. Polymorphic markers identifying haplogroups can also be relevant in marker-assisted breeding programs addressing the improvement of cluster structure and berry size.

## Competing interests

The authors declare that they have no competing interests.

## Authors’ contributions

LF, PT, LT and JMM-Z conceived the study. LLC, TL, JMB, SL and PT created and characterized the core collection. LF and GB generated and characterized the sequence data. LF, LLC, JT, AFL analysed the data. LF and JMM-Z wrote the paper with the input of all authors. All authors read and approved the final manuscript.

## Additional files

## Supplementary Material

Additional file 1: **List of the 140 individuals of the core collection used in this study.** Variety name, pedigrees when available, bred cultivars and classification according to origin and use are indicated. Classification of individuals according to K1, K2, K3 and admixed groups is described (see materials and methods section) as well as haplotype combination and corresponding haplogroups. [N.D. not determined]. Click here for file

Additional file 2: Principal component analysis of phenotypic data. Click here for file

Additional file 3: Phenotypic value presented by the 140 individuals of the core collection for the 10 traits analysed in this study. Click here for file

Additional file 4: **Distributions of the ten phenotypic traits analysed in the core collection.** Red line corresponds to the expected normal distribution. Click here for file

Additional file 5: **Genotype of the 140 core collection accessions for the 70 polymorphisms of** ***VvTFL1A.*** The standart IUB’s nomenclature for nucleic acid has been used to code SNPs. For INDEL A = deletion, C = insertion, M = heterozygous, and for triallelic microsatellites third allele has been codify as T. [n/a = not available]. Click here for file

Additional file 6: **Sequences of the 53 haplotypes found for the** ***VvTFL1A*** **gene.** Only polymorphic sites are indicated. Frequency and recombinant haplotypes are indicated. Click here for file
