# Supplementary figures and images for: Haplotype diversity of VvTFL1A gene and association with cluster traits in grapevine (V. vinifera)
Source: BMC Plant Biol. 2014 Aug 5;14:209. doi: 10.1186/s12870-014-0209-3 (PMC4243098; doi:10.1186/s12870-014-0209-3)

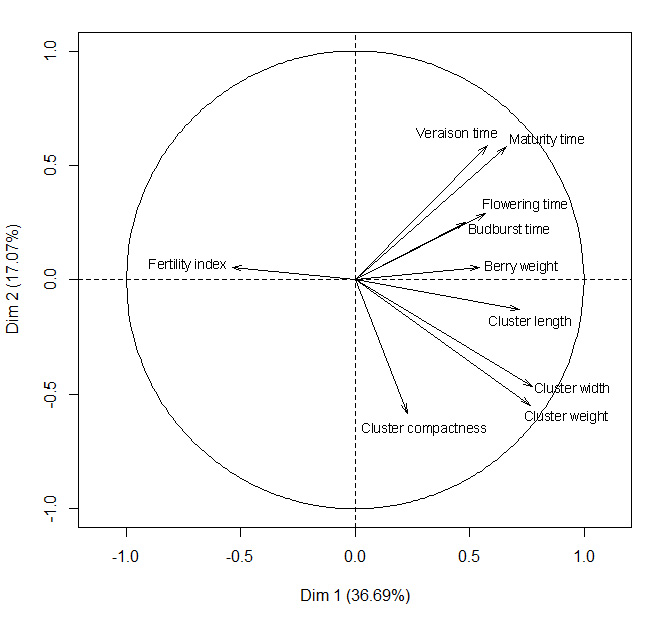

Supplement: Additional file 2: — Principal component analysis of phenotypic data. [file s12870-014-0209-3-S2.jpeg]

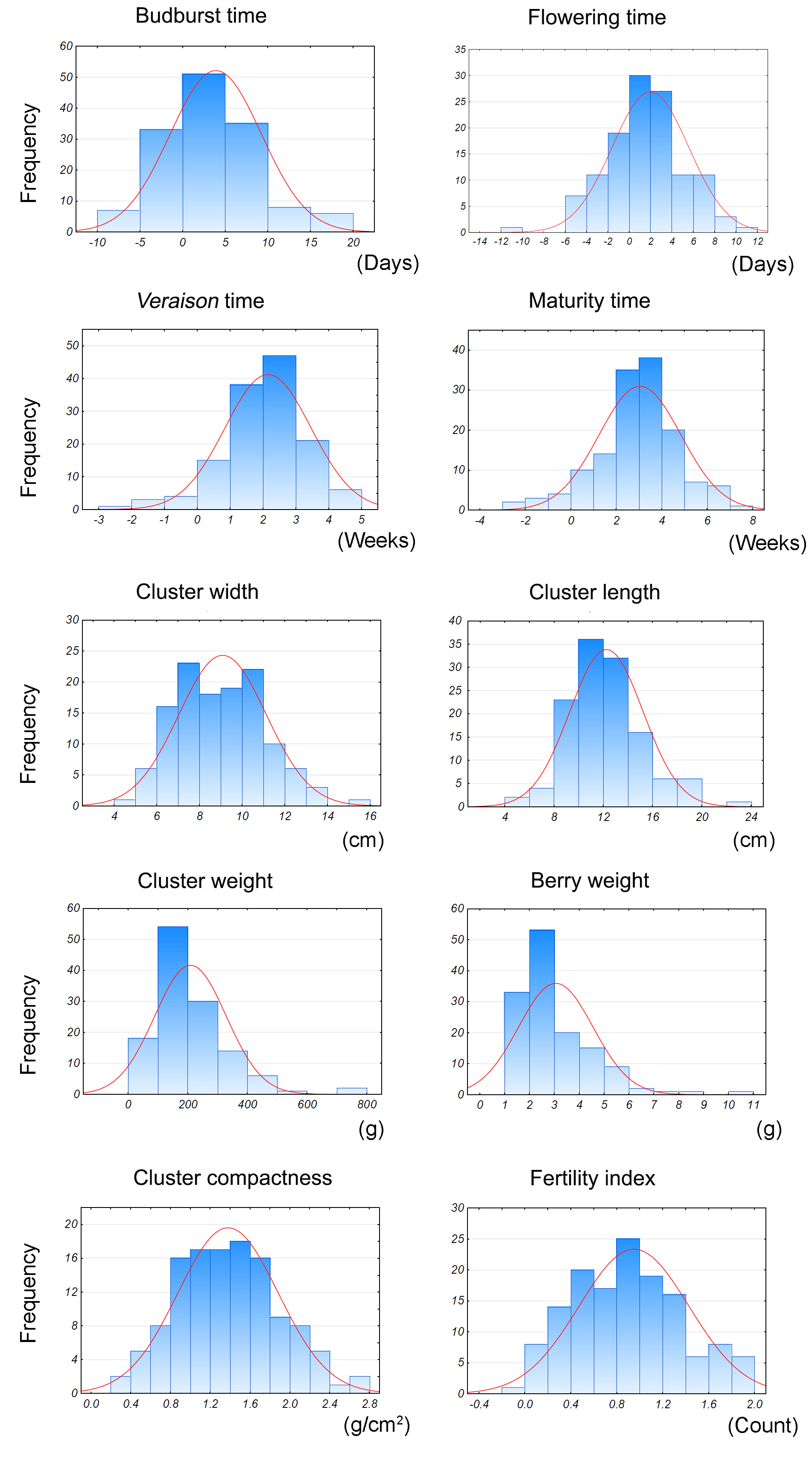

Supplement: Additional file 4: — Distributions of the ten phenotypic traits analysed in the core collection. Red line corresponds to the expected normal distribution. [file s12870-014-0209-3-S4.jpeg]
